# Fecal microbiota transplantation for ulcerative colitis: a prospective clinical study

**DOI:** 10.1186/s12876-019-1010-4

**Published:** 2019-07-04

**Authors:** Yan Tian, Yan Zhou, Sisi Huang, Jun Li, Kui Zhao, Xiaohui Li, Xiangchen Wen, Xiao-an Li

**Affiliations:** Department Of Gastroenterology, The Gastroenterology Tumor and Microenvironment Laboratory, The First Affiliated Hospital of Chengdu Medical College, Chengdu Medical College, Baoguang Road 4, Xindu district, Chengdu, 610041 Sichuan People’s Republic of China

**Keywords:** Fecal microbiota transplantation, Intestinal flora, Intestinal microorganisms, Ulcerative colitis

## Abstract

**Background:**

Fecal microbiota transplantation may contribute to disease remission in ulcerative colitis; however, the factors that determine the effects of treatment remain unknown. The aim of the present study was to prospectively investigate the clinical efficacy of fecal microbiota transplantation in patients with ulcerative colitis and identify the bacterial signatures associated with clinical remission.

**Methods:**

A total of 20 patients with ulcerative colitis were included in this prospective and uncontrolled study. All patients underwent gastroscopy five times, once every 3 weeks. Clinical indices were used to assess the efficacy of fecal microbiota transplantation, as well as the Mayo score, a score used to evaluate the extent of intestinal mucosal lesions in patients with ulcerative colitis. The changes in intestinal flora were detected by 16S ribosomal RNA-sequencing, and the relationship between ulcerative colitis and intestinal flora was analyzed.

**Results:**

After treatment, clinical index scores for diarrhea, abdominal pain, and blood stool decreased significantly (*p* < 0.05). Erythrocyte sedimentation rate and C-reactive protein levels had not changed significantly; however, the clinical index score for intestinal mucosal lesions and the Mayo score decreased significantly. In addition, 16S ribosomal RNA-sequencing revealed that the intestinal flora in patients diagnosed with ulcerative colitis was different from that of donors.

**Conclusion:**

Fecal microbiota transplantation has a potential therapeutic value for the treatment of ulcerative colitis as it changes the abundance of bacterial flora and improves the scores for diarrhea, abdominal pain, and mucous membrane lesions in patients with this disease.

**Trial registration:**

The clinical trial was retrospectively registered with ClinicalTrials.gov (NCT03016780) on January 11th, 2017.

## Background

Ulcerative colitis (UC) is a chronic and progressive intestinal inflammatory disease that can seriously affect patient quality of life. The main pathogenic mechanism of UC is thought to be aberrant activation of the immune system in response to a change in the gut environment [1–4]. However, the cause of this pathological immune system activation is not fully understood. In recent years, a growing body of evidence suggests that intestinal microorganisms may play an important role in UC pathogenesis. The species diversity of intestinal flora in patients with UC differs from that of healthy subjects. For example, patients with UC exhibit decreased intestinal populations of members of the phyla *Firmicutes* and *Bacteroidetes*, and increased populations of *Lactobacillus* [5]. In particular, the *Desulfovibrio* and *Clostridium* genera have been closely linked to UC [6]. Thus, the development of UC is closely related to changes in the intestinal flora [7–11].

Healthy intestinal flora colonizes the intestinal mucosal epithelial cells and enhances the intestinal bio-barrier function [12, 13]. The flora adheres to the surface of the intestinal mucosa to form a chemical barrier against external stimuli, which regulates intestinal immunity. However, once the homeostasis of the intestinal micro-environment is disturbed, patients are susceptible to intestinal diseases.

A range of therapies are used in the treatment of UC, including 5-aminosalicylic acid, hormone therapy, immunosuppressants, biological agents, and surgery. However, these treatments have poor efficacy. Therefore, there is a requirement for new therapeutic strategies for the treatment of UC. Fecal microbiota transplantation (FMT) may be of therapeutic value in patients with UC by contributing to the repopulation of healthy intestinal flora [14–17]. However, conflicting results have been reported regarding the efficacy of this treatment. This study evaluated clinical efficacy and safety of FMT and analyzed the relationship between UC and intestinal flora. Additionally, the effect of the intestinal flora on the intestinal mucosa was examined.

## Methods

### Study design

This prospective uncontrolled study was carried out at the Gastroenterology Department of The First Affiliated Hospital of Chengdu Medical College (Chengdu, People’s Republic of China). Patients with UC were enrolled between July 2016 and October 2017.

### Study population

In this study, 20 patients who met the UC diagnostic criteria were recruited, according to typical clinical, endoscopic, and histopathological findings. A detailed history was taken from each participant, including smoking status, disease duration, medication, and history of previous intestinal surgery.

### Inclusion and exclusion criteria

Inclusion criteria were as follows: (1) Subjects voluntarily participated in the trial and signed an informed consent form; (2) Subjects were aged 18 to 75 years, both sexes were included; (3) Subjects met the diagnostic criteria for UC; and (4) Subjects were able to communicate well with the researcher and comply with the test requirements.

Exclusion criteria were as follows: (1) Subjects were pregnant or unable to give informed consent; (2) Subjects had used immunosuppressive agents in the past 6 months; (3) Subjects had suffered of severe immunodeficiency in the previous 6 months; (4) Subjects had taken antibiotics or probiotics within the previous 6 weeks; (5) Subjects had serious complications, such as local stenosis, intestinal obstruction, intestinal perforation, toxic colon expansion, colon cancer, or rectal cancer; (6) UC was accompanied by a primary disease, such as a cardiovascular, cerebrovascular, hepatic, renal, or hematologic disease, or by a mental illness; and (7) The subject’s condition was aggravated by cessation of their normal treatment. In these cases, emergency measures were taken, the efficacy could not be judged, or the data were incomplete.

### Donor selection

Donated stool for FMT was obtained from four donors, aged between 23 and 27 years. Donors had no diagnosed medical conditions that could be potentially associated with changes in gut microbiota. Donors who had taken antibiotics or probiotics within the previous month were not included for screening. All donors were required to complete a Donor Questionnaire form. In order to prevent transmission of infectious diseases from donor to recipient, all donors underwent stool test screening [(bacterial culture and identification; fecal flora ratio examination; human rotavirus antigen determination; parasite egg detection; microscopy; serologic tests (hepatitis A virus, hepatitis B virus, hepatitis C virus, HIV antibody, syphilis, herpes simplex virus, EB virus); and *Cryptosporidium*, *Cyclospora*, and *Giardia* antigen detection)].

Every donor provided stool samples for five patients. Patients were divided into four groups, and patients from every group received stool samples from the same donor (Table 1). Clinical efficacy was compared among the four groups (Table 2). Clinical remission and response rates were calculated for all subjects after treatment.

**Table 1:** Patients’ grouping

Group1 (donor1)	Group2 (donor2)	Group3 (donor3)	Group4 (donor4)
Patient 1	Patient 2	Patient 7	Patient 14
Patient 3	Patient 5	Patient 8	Patient 15
Patient 4	Patient 6	Patient 12	Patient 18
Patient 10	Patient 9	Patient 13	Patient 19
Patient 11	Patient 16	Patient 17	Patient 20

**Table 2:** Comparison of treatment effects of different donors for patients ($\overline{\mathrm{x}}$ ± SD, *P* >0.05)

Groups	Stomach ache score	Diarrhea score	Bloody stool score	Mayo score
Group 1	1.80 ± 4.02	2.40 ± 1.3	1.20 ± 1.64	2.80 ± 2.95
Group 2	3.60 ± 3.91	1.80 ± 1.64	1.20 ± 3.42	3.40 ± 2.88
Group 3	2.40 ± 2.51	3.00 ± 3.00	0.60 ± 2.51	3.60 ± 3.21
Group 4	1.50 ± 1.73	1.50 ± 1.73	0.75 ± 1.50	1.75 ± 0.96

*P* < 0.05, the difference was statistically significant

### FMT procedure

Patients received the bowel lavage (polyethylene glycol 4000) for colonoscopy preparation the day before FMT. The median amount of donor feces was calculated (50 g) and used for FMT preparation. Donors were instructed to collect feces in a small container and to bring it to the hospital on the day of the scheduled transplant. A total of 250 mL extracted fecal suspension was prepared with 250 mL 0.9% NaCl using a conventional blender and was divided into 50 mL syringes. Filtered fecal microbiota suspension was administered into a catheter inserted into the duodenum by gastroscopy. After the procedure, the patient was returned to the ward, ensuring that their head stayed in a low position for 60 min. Following FMT, bowel movements were avoided for 0.5 h, food intake was prohibited for 1 h, and physical activity was prohibited for 2 h.

### Baseline patients’ and healthy donors’ characteristics

Patients’ and healthy donors’ baseline characteristics,including sex, age, height, weight, history of taking medicine before FMT for inflammatory bowel diseases, course of the disease, levels of inflammatory markers [erythrocyte sedimentation rate (ESR) and C-reactive protein (CRP)], colonic mucosal score, and Mayo score before FMT, are listed in Table 3.

**Table 3:** Basic patient and healthy donors’ information ($\overline{x}$ ± SD)

	Patients	Healthy donors
Sex (male/female)	20 (11/9)	4 (4/0)
Age (years)	62.50 ± 77.14	24.75 ± 0.96
Height	165.00 ± 7.90	169.50 ± 6.66
Weight	58.10 ± 9.60	62.50 ± 2.89
Drugs before treatment		
5-ASA	7	
Prednisone (combined with 5-ASA)	0	
Mesalazine suppository	2	
Course of disease (years)	4.5 (3)	
ESR (mm/h)	1.80 ± 2.52	
CRP (mg/l)	1.80 ± 2.52	
Colonic mucosal score	1.80 ± 0.70	
Mayo score	5.00 ± 2.75	

### Clinical index scores

The clinical symptom scores used to measure the efficacy of FMT were as follows: the diarrhea score, the abdominal pain score, the pus and blood stool score, the Mayo score, the bloody stool score, mucosal manifestation scoring, and colonic mucosal scoring.

Diarrhea was evaluated as follows: 0 points, no diarrhea; mild diarrhea (< 4 times/day), 3 points; moderate diarrhea (4–6 times/day), 6 points; severe diarrhea (> 6 times/day), 9 points. Abdominal pain was evaluated as follows: no abdominal pain, 0 points; mild abdominal pain, 3 points; moderate abdominal pain (4–6 times/day), 6 points; severe abdominal pain, 9 points. Pus and blood in stool were evaluated as follows: no pus and blood in stool, 0 points; mild pus and blood, 3 points; moderate pus and blood, 6 points; severe pus and blood, 9 points.

The Mayo score was used to evaluate the extent of intestinal mucosal lesions in UC. The scoring was as follows: normal stool frequency, 0 points; more than normal stool frequency (1–2 times/day), 1 point; more than normal stool frequency (3–4 times/day), 2 points; very high (more than 5 times/day), 3 points.

Bloody stool was evaluated as follows: no blood in the stool, 0 points; a little blood in the stool, 1 point; obvious bloody stool, 2 points; mostly bloody stool, 3 points. The mucosal manifestation scoring was categorized as follows: normal mucosa, 0 points; mild fragility, 1 point; moderate fragility, 2 points; moderate fragility with exudation, 3 points. The colonic mucosal scoring criteria were as follows: normal intestinal mucosa, 0 points; mucosal congestion and blood vessel blushing, 1 point; mucosal contact bleeding, 2 points; mucosal spontaneous bleeding, 3 points; and mucosal ulcers, 4 points.

Finally, the ESR and CRP levels were used as indicators of inflammatory reactivity. An automatic ESR analyzer was used to detect ESR, and transmitted immunoturbidimetric method was used to determine CRP at the laboratory of the First Affiliated Hospital of Chengdu Medical College.

### Intestinal flora analysis

16S ribosomal RNA sequencing (16S rRNA-seq) analysis was performed on the bacterial rRNA from stool of healthy donors and patients with UC before treatment and after the first and second treatment (groups d0, d1, and d2). DNA was first extracted from the stools of healthy donors and patients. DNA pre-amplification and sequencing was carried out by Tianjin Novo Zhiyuan.

First, PCR pre-amplification was conducted to determine whether the samples met the quality control criteria. The DNA primer sequences were 341-F: (5′-CCTACACGACGCTCTTCCGATCTN-3′) and 805-R: (5′-GACTGGAGTTCCTTGGCACCCGAGAATTCCA-3′). Barcode-tagged primers were used to perform PCR amplification of the 16S V3-V4 region. The products were subjected to quality inspection, purification, and library construction. After the alignment, 16S rRNA-sequencing was performed on the Illumina Hiseq-PE250 technology sequencing platform, using the double-end sequencing method, resulting in 3,043,659 high-quality sequences. Next, the relevant statistical analyses were performed.

### 16S rRNA-seq analysis

After removal of the chimeras, the filtered high-quality sequences were grouped into 8650 operational taxonomic units (OTUs). In all samples, 99.962 and 67.5% of the total sequence were assigned to 14 and 142 genera, respectively. Unclassified bacteria accounted for approximately 0.038% of the total sequence (Tables 4 and 5).

**Table 4:** Quality of 16S rRNA sequencing of fecal bacteria in donors

Sample ID	Raw Reads	Combined Raw Tags	Qualified Raw Tags	Effective Base (nt)	Q30(%)	GC(%)	Effective(%)
Healthy 1	97,968	87,682	75,161	28,871,200	96.67	50.96	71.22
Healthy 1	95,320	85,873	73,759	28,455,303	96.76	51.00	72.11
Healthy 1	99,573	89,409	75,142	29,248,027	96.66	50.79	70.43
Healthy 1	91,806	82,526	70,806	26,899,361	96.66	50.79	70.83
Healthy 2	88,237	79,622	66,615	26,326,275	96.52	52.50	71.68
Healthy 2	92,061	82,663	70,466	27,102,744	96.64	51.35	71.16
Healthy 2	94,606	84,899	72,022	27,386,297	96.53	52.06	69.92
Healthy 2	83,508	74,456	61,895	24,076,713	96.40	52.20	69.32
Healthy 3	97,962	86,811	69,050	25,770,148	94.35	51.31	63.31
Healthy 3	88,333	79,683	65,667	23,865,511	94.86	51.37	65.25
Healthy 3	97,891	86,659	68,235	25,726,245	94.07	51.15	62.98
Healthy 3	94,621	83,569	65,945	24,366,516	94.30	51.87	62.11
Healthy 4	97,662	86,481	67,588	25,200,871	94.21	51.56	62.19
Healthy 4	94,153	83,751	66,134	24,357,916	94.09	51.81	62.32
Healthy 4	86,745	77,552	62,184	22,912,891	94.25	51.40	63.72
Healthy 4	80,899	71,599	56,065	21,052,071	94.23	50.96	62.33

**Table 5:** The quality of 16S rRNA sequencing of fecal bacteria in patients with ulcerative colitis

Sample ID	Raw Reads	Combined Raw Tags	Qualified Raw Tags	Effective Bases (nt)	Q30 (%)	GC (%)	Effective(%)
d0-A3	93,040	88,487	79,811	30,944,428	97.16	52.49	80.8
d0-A5	83,603	79,229	70,709	27,506,095	97.19	51.77	78.56
d0-A6	97,570	91,686	82,546	31,275,447	97.18	50.58	76.69
d0-A8	86,502	81,642	73,450	27,695,120	97.17	51.43	76.82
d0-A10	81,604	77,275	70,049	26,399,958	97.15	51.3	78.02
d0-A11	98,275	92,745	83,720	32,698,787	97.13	51.45	79.48
d0-A12	85,580	77,340	60,971	22,690,822	94.2	51.9	63.55
d0-A14	89,314	84,745	76,866	28,211,684	97.22	52	76.51
d0-B1	91,830	87,008	76,747	30,617,125	96.95	53.2	79.9
d0-B2	82,081	77,921	67,290	27,251,920	96.81	54.73	77.99
d0-B4	91,046	86,561	77,753	30,224,143	97.1	53.54	80.6
d0-B7	88,594	83,843	72,949	28,241,953	96.94	52.89	75.45
d0-B9	89,766	85,407	77,936	28,937,019	97.21	51.59	78.3
d1-A3	94,602	89,947	80,540	31,277,847	97.13	52.55	80.08
d1-A5	88,810	83,587	75,024	29,506,841	97.12	51.8	79.36
d1-A6	92,458	87,430	78,588	30,928,414	97.13	51.74	80.44
d1-A8	88,997	83,816	74,889	28,211,660	97.14	51.58	75.6
d1-A10	95,021	89,688	80,983	30,012,662	97.09	51.25	76.24
d1-A11	88,286	83,310	75,278	28,145,749	97.16	51.5	76.35
d1-A12	97,742	88,876	68,748	26,725,630	94.22	53.22	65.16
d1-A13	86,350	81,399	72,066	27,885,218	97.04	52.02	76.87
d1-B1	84,009	79,994	72,638	27,442,698	97.22	52.26	79.48
d1-B2	92,523	88,004	76,896	31,318,550	96.88	54.09	79.79
d1-B4	99,557	94,523	83,760	33,197,434	97.05	52.82	79.67
d1-B7	81,447	76,527	68,433	25,102,970	97.1	51.84	73.74
d1-B9	85,819	81,046	72,942	28,034,477	97.07	51.26	78.23
d2-A8	86,695	82,017	73,730	26,868,038	97.15	52.25	74.5
d2-A12	82,621	78,323	69,553	26,127,370	97.04	51.21	75.78
d2-B2	85,964	81,497	71,186	28,547,029	96.92	53.42	78.78
d2-B7	81,000	76,000	67,820	24,654,602	97.05	51.4	72.8

Summary of columns:

(1) Sample ID: sample name

(2) Raw Reads: the number of PE-Reads in the original machine

(3) Combined Raw Tags: the spliced tags sequence

(4) Qualified Raw Tags: tags sequence for subsequent analysis after quality control filtering

(5) Effective Bases (nt): the number of bases of the effective tags

(6) Q30 (%): the percentage of bases with a base quality value greater than 30 (sequencing error rate < 0.1%) in effective tags

(7) GC (%): the content of GC base in the effective tags

(8) Effective Rate (%): The ratio of effective tags to raw PE-reads

### Statistical analysis

Measurement indicators are represented as mean and standard deviation, and counting indicators are presented as number and percentage of each category. The intra-group comparison of measurement indicators was performed using paired t-test or a Wilcoxon’s signed-rank test. The count index was tested using a paired chi-squared (χ^2^) test, and the grade index was tested using a Wilcoxon’s signed-rank test. *P*-values < 0.05 were considered statistically significant.

## Results

### Patient characteristics

This study included 11 men and 9 women aged from 18 to 73 years. All patients completed five rounds of FMT treatment, once every 3 weeks.

### Clinical outcomes

The diarrhea score showed a downward trend after treatment, as shown in Table 6 (3.75 ± 3.49 before treatment; 0.79 ± 1.69 after the fifth treatment). The mean diarrhea score was significantly decreased following five rounds of FMT, as compared to the diarrhea score before treatment. The abdominal pain score also showed a significant downward trend after treatment, as shown in Table 6 (2.55 ± 2.63 before treatment; 1.42 ± 1.54 after the fourth treatment). The bloody stool score also showed a downward trend after treatment, as shown in Table 6 (3.30 ± 2.36 before treatment; 0.79 ± 1.36 after the fifth treatment).

**Table 6:** Symptom scores of ulcerative colitis patients before and after five rounds of FMT ($\overline{x}$ ± SD; *P* < 0.05)

Time	Diarrhea score	Stomach ache score	Bloody stool score
Before treatment	3.75 ± 3.49	2.55 ± 2.63	3.30 ± 2.36
After the first treatment	2.55 ± 2.63	1.80 ± 1.79	2.25 ± 2.15*
After the second treatment	2.21 ± 2.62	1.26 ± 1.52	1.74 ± 1.82*
After the third treatment	1.42 ± 2.52*	1.42 ± 1.54*	0.95 ± 1.43*
After the forth treatment	1.26 ± 2.31*	1.42 ± 1.54*	1.11 ± 1.49*
After the fifth treatment	0.79 ± 1.69*	0.63 ± 1.26	0.79 ± 1.36*

*P < 0.05, the difference was statistically significant

**Fig. 1:**
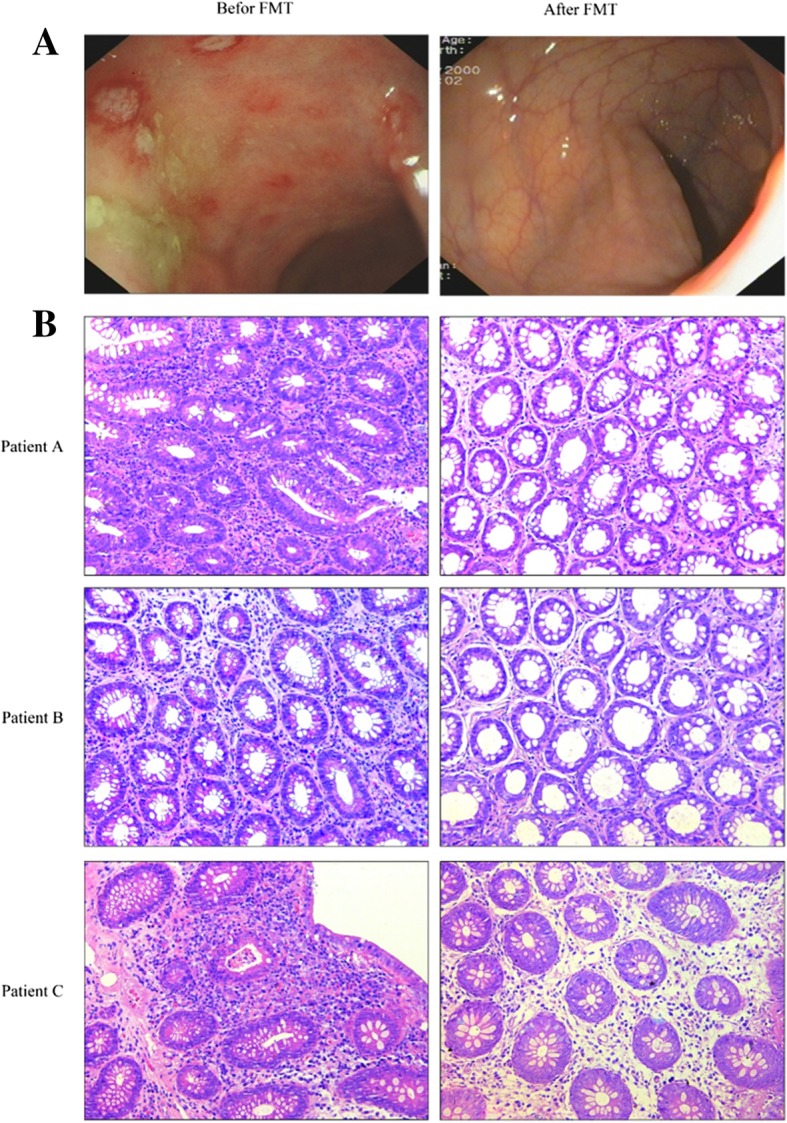
Enteroscopic findings of intestinal mucosa of a patient before and after treatment (**a**); Hematoxylin-eosin staining of intestinal mucosa before and after treatment (**b**);**b** reflect the infiltration of inflammatory cells in the intestinal mucosa before and after treatment. PatientA, PatientB, PatientC represent three patients. Scale bar = 200 μm

The endoscopic intestinal mucosal score and the Mayo score were used to evaluate the intestinal mucosa and disease activity, respectively, in patients with UC before and after FMT. Post-treatment, the intestinal mucosal score (1.37 ± 0.60) was significantly lower than that at the pre-treatment time point (1.80 ± 0.70, *p* < 0.05). Likewise, the post-treatment Mayo score (3.00 ± 2.00) was significantly lower than the pre-treatment Mayo score (5.00 ± 2.75) (Table 7 and Fig. 1). Compared with pre-treatment, The aspect of the intestinal mucosal under enteroscopy improved and the infiltration of inflammatory cells in the intestinal mucosa decreased after FMT (Fig. 1a and b).

**Table 7:** Mayo and colonic mucosal scores in ulcerative colitis patients before and after five rounds of FMT $\Big(\overline{x}$ ± SD; *P* < 0.05)

Time	Colonic mucosal score	Mayo score
Before treatment	1.80 ± 0.70	5.00 ± 2.75
After the fifth treatment	1.37 ± 0.60*	3.00 ± 2.00*

*P < 0.05, the difference was statistically significant

The erythrocyte sedimentation rate (ESR) and C-reactive protein (CRP) were used as indicators of inflammatory reactivity. These measures did not change significantly following FMT treatment (Table 8).

**Table 8:** Erythrocyte sedimentation rate (ESR) and C-reactive protein levels (CRP) in patients with ulcerative colitis before and after five rounds of fecal microbiota transplantation $\Big(\overline{x}$ ± SD; *P* > 0.05)

Time	ESR (mm/h)	CRP (mg/L)
Before treatment	12.17 ± 12.44	1.80 ± 2.52
After the fifth treatment	11.63 ± 10.71	1.73 ± 2.14

*P* < 0.05, the difference was statistically significant

### Safety evaluation

No patient had serious adverse reactions during the study period and follow-up period. One patient developed skin erythema on the first day after treatment. We consider that it is related to allergic reaction, the erythema disappeared quickly after anti-allergy treatment. Other three patients had mild abdominal distension that resolved without clinical treatment within 24 h.

### Analysis results of intestinal flora

#### Relative abundance of intestinal flora in patients with UC and healthy donors

**Fig. 2:**
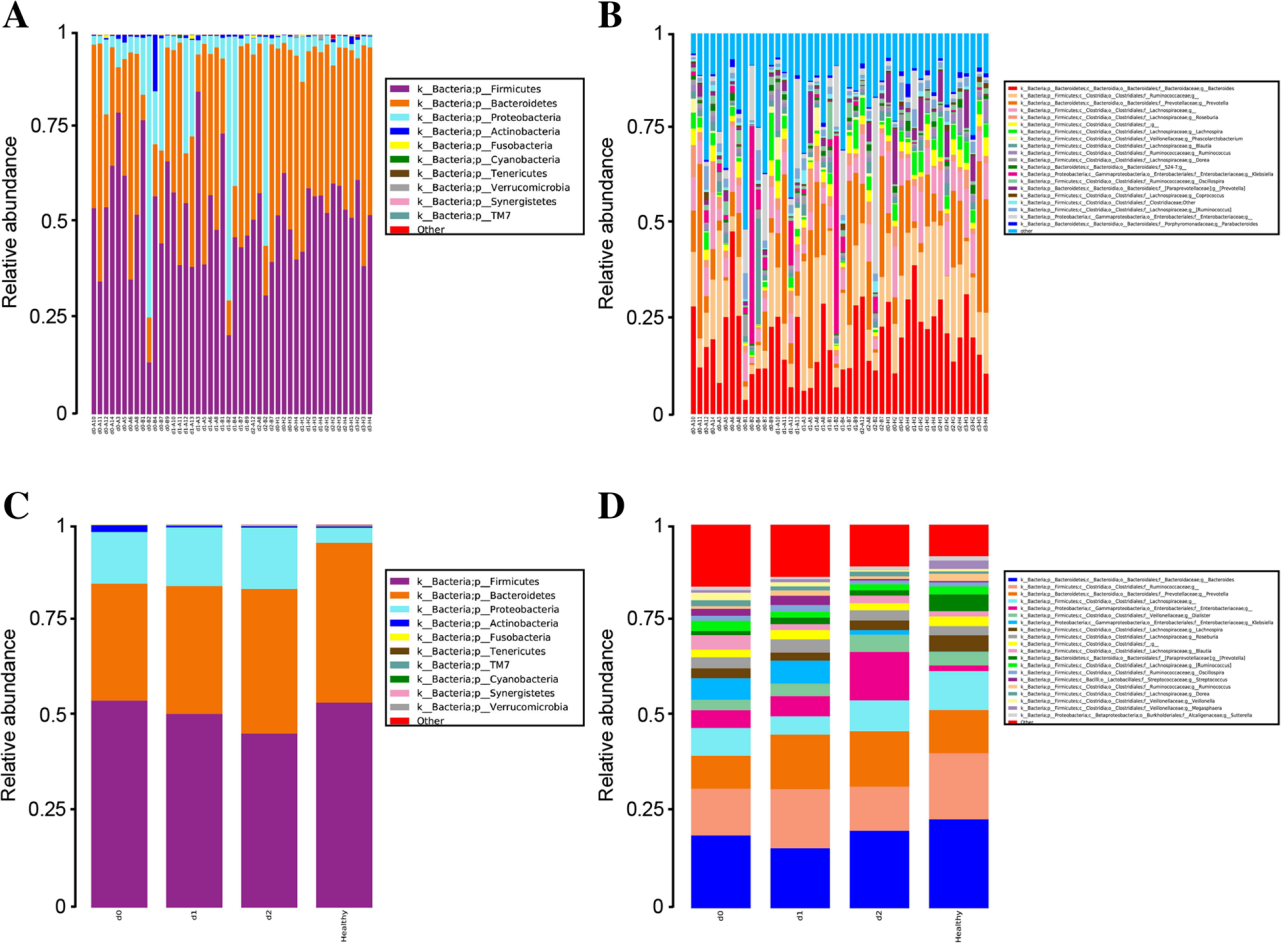
Relative abundance of fecal microbiota in patients with ulcerative colitis before and after treatment at phylum and genus level. Different colors represent different bacterial species. **a** 10 most abundant species in patients and donors at the phylum level; **b** 20 most abundant species in patients and donors at the genus level; **c** 10 most abundant species in patients at the phylum level according to different treatment stages; **d** 20 most abundant species in patients at the genus level according to different treatment stages

ANOVA and LSD statistical analyses revealed no significant difference in intestinal microbial composition in patients with UC versus healthy donors at all intestinal levels. At the phylum level, all samples were dominated by *Bacteroidetes*, *Firmicutes*, and *Proteobacteria*, which totally accounted for 98.9% of the sequence reads. Bacteria present at lower proportions included *Actinobacteria*, *Fusobacteria*, and *Verrucomicrobia*, accounting for 0.8, 0.1, and 0.1% of the total sequence reads, respectively (as shown in Fig. 2a). At the genus level, all samples were dominated by *Bacteroides, Prevotella*, *Ruminococcaceae*, and *Lachnospiraceae*, accounting for 19.5, 14.7, 11.9, and 7.5% of the total sequence reads, respectively. In addition, *Enterobacteriaceae*, *Klebsiella*, *Roseburia*, *Clostridiales*, *Lachnospira*, and *Blautia* were also relatively abundant, accounting for 4.3, 3.4, 2.8, 2.3, 3.0, and 2.1% of the total sequence reads, respectively. The lesser abundant genera include *Dorea*, *Parabacteroides*, and *Coprococcus*, accounting for 1.0, 0.7, and 0.5% of the total sequence reads, respectively (as shown in Fig. 2b).

Although no significant differences in gut microbiota composition were found at the phylum level, a significant change in the composition of the flora was detected in patients with UC compared to healthy donors. We analyzed the composition of intestinal flora before treatment (d0) and after the first (d1) and second (d2) treatments, and found that at the level of the phylum, the proportion of *Firmicutes* in the d0 stage was higher, accounting for 54.0% of the total sequence reads, which was similar to the proportion of the donor group. The proportion of *Firmicutes* showed a downward trend after treatment in the group of patients with UC. *Bacteroidetes* in the donor group accounted for 41.8% in the healthy donor group, and accounted for 30.5, 33.4, and 37.8% in the d0, d1, and d2 groups, respectively. This gradual upward trend indicates that the relative abundance of *Bacteroidetes* gradually approached that of the donor group after treatment. Furthermore, the relative abundance of *Proteus* in the d0, d1, and d2 groups was significantly higher than that of the healthy donor group, accounting for 13.5, 15.3, and 16.0%, respectively (as shown in Fig. 2c). At the genus level, the proportion of *Bacteroides* in the d0, d1, and d2 groups was 18.9, 15.6, and 20.2%, respectively; being lower than in the healthy donor group. *Prevotella* proportions in d0, d1, and d2 groups was lower than that of donor group and accounted for 8.6, 14.2, and 14.5% of sequence reads, respectively. *Klebsiella* abundance in the d0, d1, and d2 groups was 5.6, 5.9, and 1.2%, respectively, while that of the donor group was only 0.2%. In the d0, d1, and d2 groups, *Streptococcus* accounted for 1.8, 2.4, and 0.4%, respectively, compared to that of the donor group, which was 0.3%. The relative abundance of *Streptococcus* before treatment was significantly higher than that of the healthy donor group and decreased significantly in the d2 group (as shown in Fig. 2d).

#### Venn diagram analysis results

**Fig. 3:**
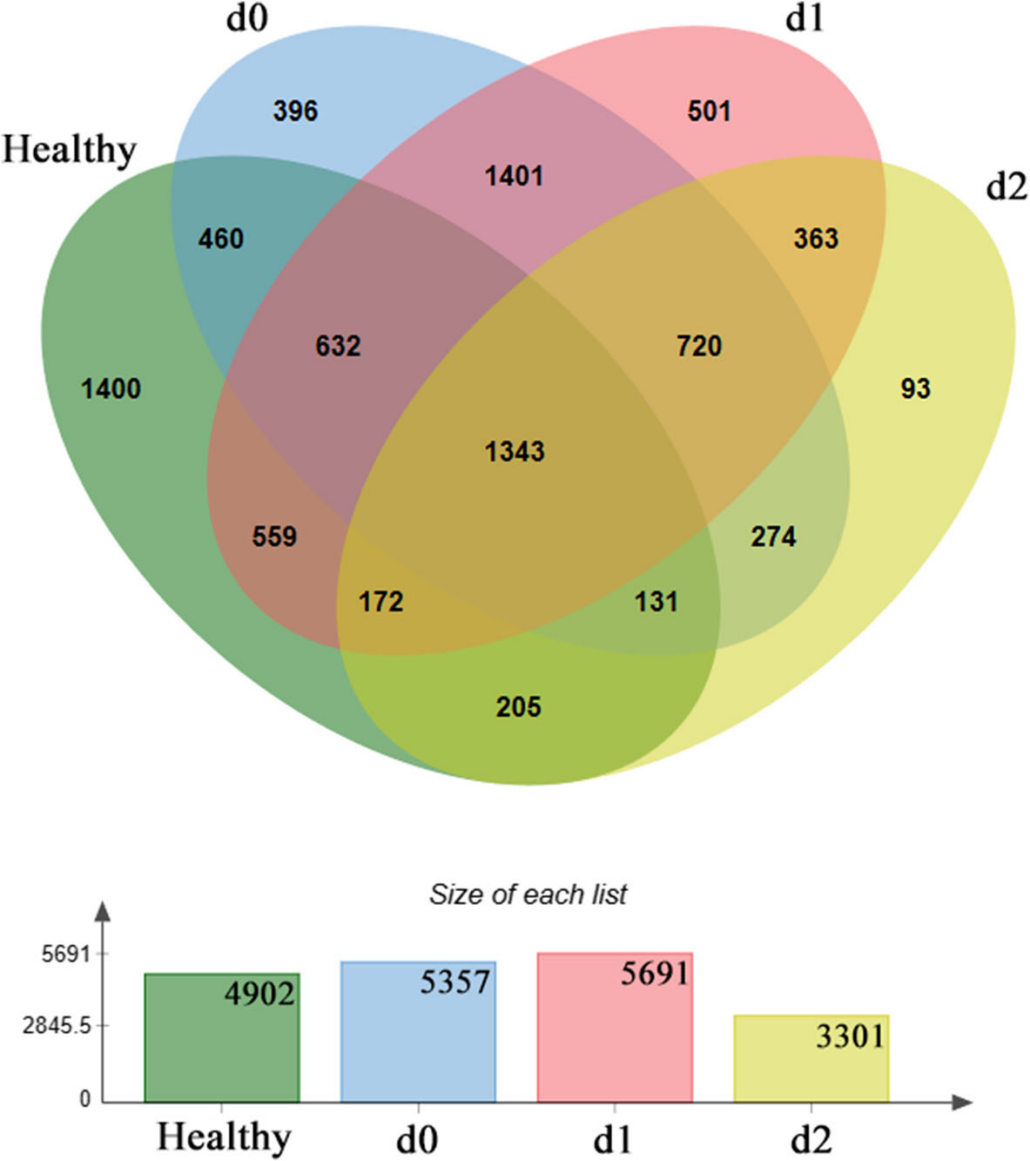
Venn diagram analysis of bacteria populations in patients with ulcerative colitis and healthy donors. The number in each region represents the number of operational taxonomic units shared between sample groups or unique to the sample group. The histogram represents the total number within each sample set

The Venn diagram reflects the overlap between OTU at different treatment stages, with a large number of overlapping OTUs in each treatment stage. A total of 1342 OTUs were found. The most highly contrasting OTU was that between the patient group d0 and the healthy donor group. However, following treatment, there was an increase in the number of overlapping strains and a decrease in the number of unique OTUs between the healthy donor group and d1 and d2 groups (Fig. 3).

#### Diversity analysis results of intestinal bacterial populations

**Fig. 4:**
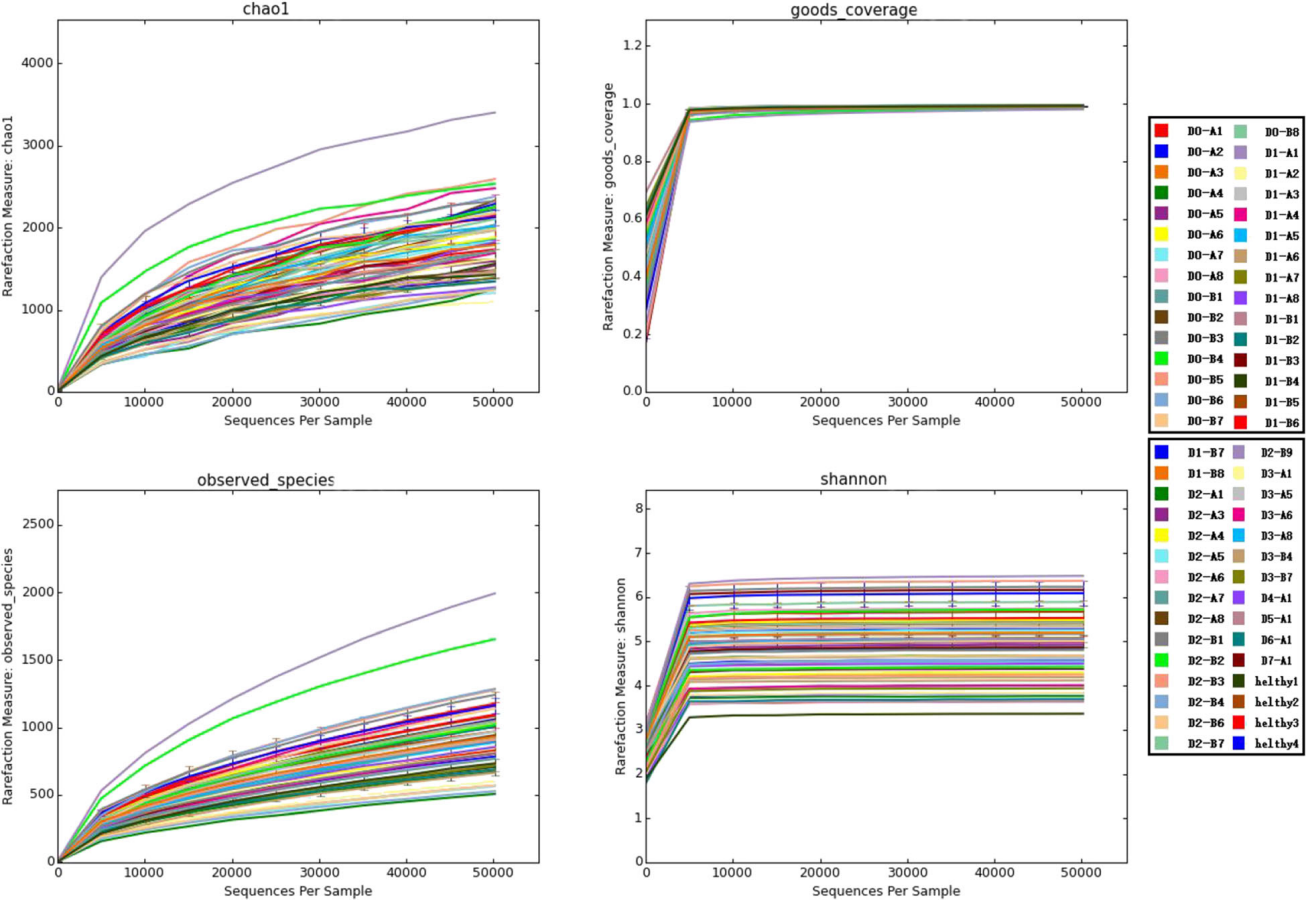
Dilution curve of alpha-diversity. The alpha-diversity index based on the number of sequencing reads. Different samples are represented by different color lines

**Fig. 5:**
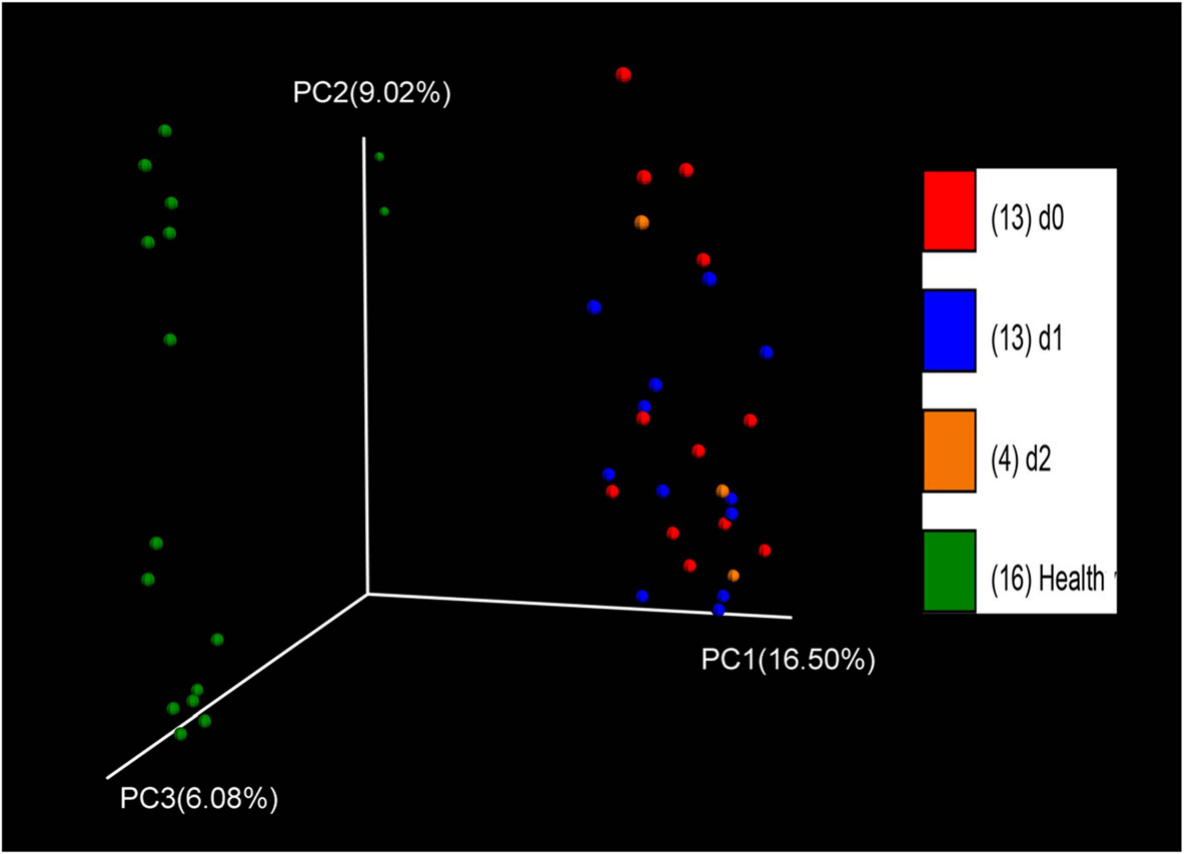
Principal component analysis of donors and patients with ulcerative colitis during each treatment period. PC1, PC2, and PC3 represent the three principal components, and the percentage indicates the contribution of the principal component to the sample difference. Each dot represents one sample, and each color represents one group

Alpha diversity is used to measure the species diversity of a single ecological sample of a community, and is a comprehensive indicator reflecting the richness and uniformity of a population. Alpha diversity accounts for the following factors: observed species index, the Chao I index, and the Shannon index. A dilution curve of goods-coverage reflects whether the sequencing results are an accurate representation of the sample. Figure 4 shows the alpha diversity dilution curve of each sample. The dilution curve of goods-coverage is close to the plateau stage, indicating that the sequencing amount of this test is close to saturation. This indicates that the number of sequencing reads closely reflects the diversity composition of the fecal flora of each sample in this experiment. The sequencing depth is sufficient. Judging by the OTU number, the Shannon index and the Chao I index, the bacterial diversity in the stool samples of patients with UC decreased with the number of treatments. However, this decrease was not statistically significant. The change in the bacterial composition of the stool samples was related to altered bacterial colonization of the intestine (Fig. 4). Beta diversity reflects the similarity of microbial communities. The bacterial community clustering remained unchanged after FMT in patients with UC (Fig. 5).

#### LDA effect size analysis results

**Fig. 6:**
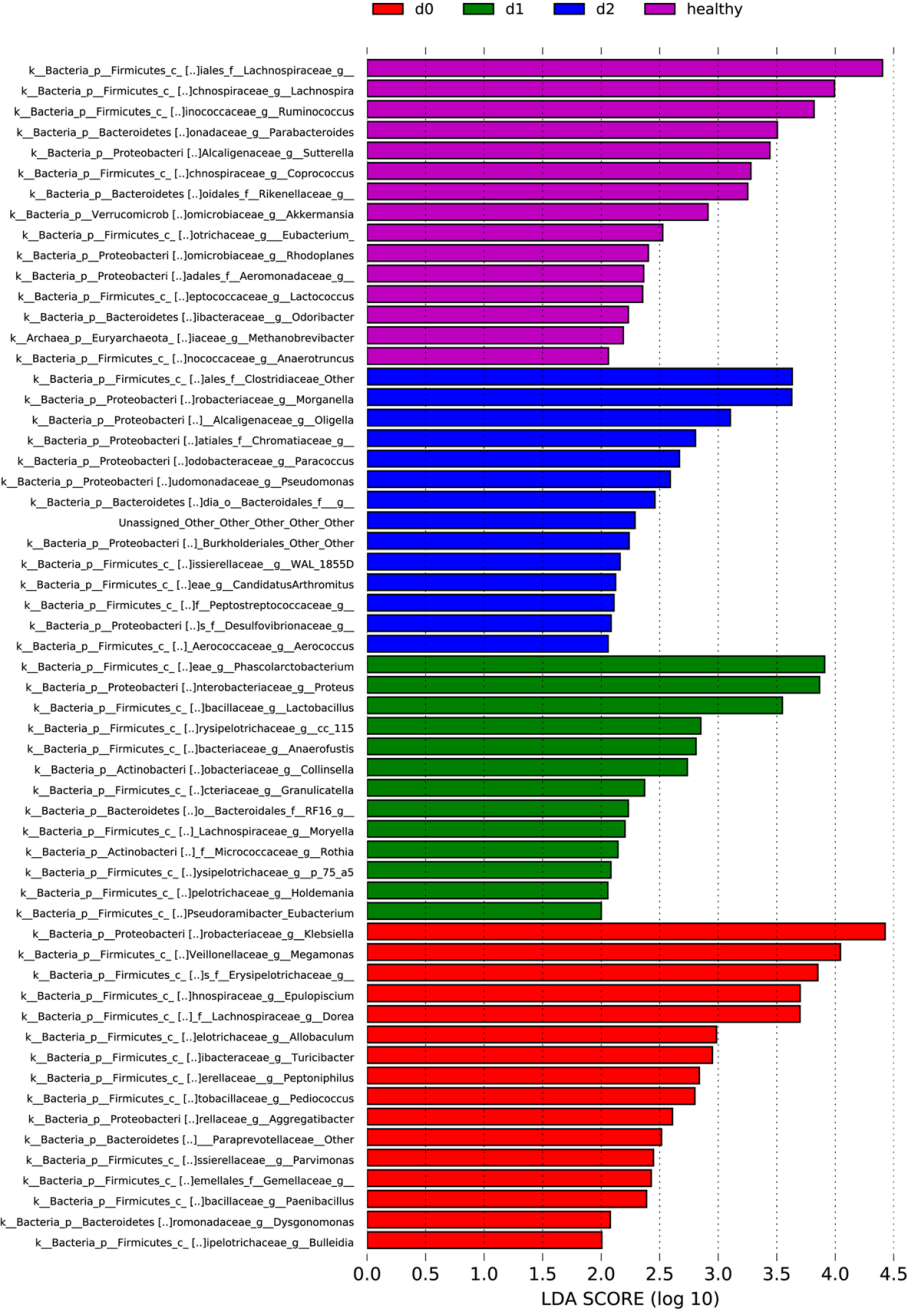
Linear discriminant analysis (LDA) scores of donors and patients with ulcerative colitis during each treatment period (for specimens with an LDA score > 2)

LDA Effect Size (LEFSe) is used to analyze species that have significant differences in abundance between groups (biomarkers). As shown in Fig. 6, a total of 58 significantly different genera were identified between the donor healthy group and the group of patients with UC by LEFSe. Among them, the intestinal bacterial populations of the healthy donor group were dominated by *Lachnospiraceae*, *Ruminococcus*, *Parabacteroides*, *Sutterella*, and *Akkermansia*, and that of the patients in group d0 was dominated by *Klebsiella*, *Megamonas*, *Erysipelotrichaceae*, *Epulopiscium*, and *Dorea*. The intestinal bacterial populations of group d1 were dominated by *Phascolarctobacterium*, *Proteus*, and *Lactobacillus*, and that of the group d2 was dominated by *Clostridiaceae* (Fig. 6).

## Discussion

This study compared changes in abdominal pain scores, diarrhea scores, bloody stool scores, endoscopic intestinal mucosal score, and Mayo scores before and after FMT treatment in patients with UC and evaluated the clinical efficacy of FMT in the treatment of UC. The results showed that the patients’ abdominal pain score, diarrhea score, bloody stool score, intestinal mucosal lesion, and Mayo score significantly decreased after treatment. This is consistent with previous research results [18].

However, CRP level and ESR did not significantly change following FMT treatment. This suggests that FMT can improve the clinical symptoms and mucosal lesions, reduce disease activity, and slightly reduce the disease inflammatory response in patients with UC.

The earliest application of FMT for the treatment of UC was attempted in 1988, and showed that patients’ UC symptoms significantly improved [19]. Brandt et al. [20] followed up 6 patients with UC after FMT treatment and also found that their symptoms were alleviated. Kump et al. [21] found that FMT improves the clinical symptoms of patients with UC by regulating intestinal flora. Our results also showed that FMT improves abdominal and bowel discomfort symptoms in patients with UC.

The intestinal microenvironment plays an important role in maintaining the intestinal mucosal immunity and regulating the intestinal function. Here, we found that in both patients with UC and healthy donors, intestinal flora was mainly composed of *Bacteroidetes*, *Firmicutes*, and *Proteobacteria*. However, the ratio of *Bacteroides* to *Proteobacteria* was significantly different between patients with UC and healthy donors, which is consistent with the findings of previous reports [22].

At the genus level, the relative abundance of *Prevotella* before treatment was lower than that of the donor group. The relative abundance of *Klebsiella* and *Streptococcus* was higher than that of the donor group. After treatment, the relative abundance of these three genera gradually became similar to that of the health donors. Therefore, the decrease of *Prevotella*, and the increase of *Klebsiella* and *Streptococcus* proportions may be important factors leading to the onset of UC.

Indeed, LEfSe analysis indicated that there was a difference in the intestinal flora between donor and patients. Samples from the d0 group of patients with UC were dominated by *Klebsiella*, *Megamonas*, *Erysipelotrichaceae*, *Epulopiscium*, and *Dorea*. These genera may be related to the pathogenesis of UC, and this information may therefore be of clinical value for improving the diagnosis of UC.

Furthermore, we carried out an OTU-based Venn diagram analysis and found that the number of overlapping OTUs between patients with UC in different treatment stages and donor groups increased gradually, indicating a gradual return of the patient intestinal flora to the healthy state. Furthermore, the number of non-overlapping OTUs gradually decreased. This indicates that FMT can, to a certain extent, correct UC-associated dysbiosis. Due to the clinical efficacy of FMT treatment, it can be speculated that these dominant bacteria may improve the symptoms of patients with UC, but this hypothesis requires further verification.

Our findings showed that FMT is a safe and effective treatment for UC. After transplantation, the symptoms of diarrhea, abdominal pain, and bloody stools improved. Intestinal mucosal lesions improved, and the Mayo score decreased. Furthermore, we have shown that FMT can regulate intestinal flora. Therefore, FMT can be used as a novel therapy for the treatment of UC. However, if used widely in a clinical setting, the FMT procedure must be standardized (e.g., donor selection, stool preparation, delivery route, and dosing). Therefore, there is a requirement for further evidence from long-term and randomized controlled clinical studies examining donor and recipient microbiota composition.

Our study has some limitations. First, the number of patients that we have recruit is not too much;Second, the study performed in a single institution,this may limite the Diversity of study. So, other more perfect study is necessary in the futuer.

## Conclusion

Fecal microbiota transplantation improves symptoms in patients with UC through changing the abundance of bacterial flora. This study provides a valuable treatment modality for UC.

## Data Availability

The datasets used and/or analyzed during the current study are available from the corresponding author on reasonable request.

## Abbreviations

16S rRNA-seq: 16S Ribosomal RNA-sequencing
CRP: C-reactive protein
ESR: Erythrocyte sedimentation rate
FMT: Fecal microbiota transplantation
LEFSe: LDA Effect Size
OTU: Operational taxonomic unit
UC: Ulcerative colitis
